# Racial Differences in Clinical Outcomes for Metastatic Renal Cell Carcinoma Patients Treated With Immune-Checkpoint Blockade

**DOI:** 10.3389/fonc.2021.701345

**Published:** 2021-06-16

**Authors:** T. Anders Olsen, Dylan J. Martini, Subir Goyal, Yuan Liu, Sean T. Evans, Benjamin Magod, Jacqueline T. Brown, Lauren Yantorni, Greta Anne Russler, Sarah Caulfield, Jamie M. Goldman, Wayne B. Harris, Omer Kucuk, Bradley C. Carthon, Viraj A. Master, Bassel Nazha, Mehmet Asim Bilen

**Affiliations:** ^1^ Department of Hematology and Medical Oncology, Emory University School of Medicine, Atlanta, GA, United States; ^2^ Winship Cancer Institute of Emory University, Atlanta, GA, United States; ^3^ Departments of Biostatistics and Bioinformatics, Emory University, Atlanta, GA, United States; ^4^ Department of Pharmaceutical Services, Emory University School of Medicine, Atlanta, GA, United States; ^5^ Department of Urology, Emory University School of Medicine, Atlanta, GA, United States

**Keywords:** renal cell carcinoma, immunotherapy, immune-checkpoint-inhibitors, racial disparities, real-world outcomes, anti-PD-1/PD-L1, disparities (health racial)

## Abstract

**Background:**

Immune-checkpoint-inhibitors (ICIs) have become the cornerstone of metastatic renal-cell-carcinoma (mRCC) therapy. However, data are limited regarding clinical outcomes by race. In this study, we compared the real-world outcomes between African American (AA) and Caucasian mRCC patients treated with ICIs.

**Methods:**

We performed a retrospective study of 198 patients with mRCC who received ICI at the Emory Winship Cancer Institute from 2015-2020. Clinical outcomes were measured by overall survival (OS), progression-free survival (PFS), and overall response rate (ORR) defined as a complete or partial response maintained for at least 6 months per response evaluation criteria in solid tumors version 1.1. Univariate and multivariable analyses were carried out for OS and PFS by Cox proportional-hazard model and ORR by logistical-regression model. Descriptive statistics compared rates of immune-related adverse events (irAEs) and non-clear-cell-RCC (nccRCC) histology were assessed using Chi-square test.

**Results:**

Our cohort was comprised of 38 AA and 160 Caucasian patients. Most were diagnosed with clear-cell-RCC (ccRCC) (78%) and more than half received (57%) PD-1/PD-L1 monotherapy. Most patients were intermediate or poor-risk groups (83%). Comparing to Caucasians, our AA cohort contained more females and nccRCC cases. Kaplan-Meier method showed AAs had no statistically different median OS (17 *vs* 25 months, p=0.368) and PFS (3.1 *vs* 4.4 months, p=0.068) relative to Caucasian patients. On multivariable analysis, AA patients had significantly shorter PFS (HR=1.52, 95% CI: 1.01-2.3, p=0.045), similar ORR (OR=1.04, 95% CI: 0.42-2.57, p=0.936) and comparable OS (HR=1.09, 95% CI: 0.61-1.95, p=0.778) relative to Caucasians.

**Conclusions:**

Our real-world analysis of ICI-treated mRCC patients showed that AAs experienced shorter PFS but similar OS relative to Caucasians. This similarity in survival outcomes is reassuring for the use of ICI amongst real-world patient populations, however, the difference in treatment response is poorly represented in early outcomes data from clinical trials. Thus, the literature requires larger prospective studies to validate these findings.

## Introduction

Immune checkpoint inhibitors (ICIs) are now a major treatment option for metastatic renal cell carcinoma (mRCC). There have been numerous agents developed including Programmed Death Receptor-1 (PD-1: Nivolumab, Pembrolizumab), Programmed Death Receptor Ligand-1 (PDL-1: Atezolizumab) and Cytotoxic T-lymphocyte Protein-4 blockers (CTLA-4: Ipilimumab) (1, 2). In clinical trials, ICI monotherapy and combination therapies have displayed improved efficacy and favorable toxicity profiles for mRCC patients relative to the older regimens (3–5). However, patients of racial and ethnic minorities were underrepresented in the ICI clinical trials that led to the regulatory approval of these agents in several tumor types, including mRCC (6). For instance, only 5 AA patients were enrolled in the 821 patient CHECKMATE-025 trial comparing nivolumab to everolimus in mRCC patients receiving prior standard of care treatment (7). A study that compared the demographics of RCC clinical trials to the overall RCC patient population found that AAs made up less than 7% of the clinical trial samples despite comprising nearly 10% of the population with disease (PWD) (8). Researchers have identified numerous reasons for the poor participation of certain minority groups in clinical trials citing both structural and patient-specific factors such as age, socioeconomic status, financial barriers, culturally based mistrust of medical institutions and medical comorbidities (9).

The major classification schema for RCC exists between the predominating clear cell and non-clear cell histology. NccRCC makes up the minority of patients comprising 20-25% of all RCC diagnosis (10). The nccRCC pathophysiology does not show a clear correlation to the well-studied Von Hippel Lindau (VHL) pathway that develops ccRCC and, thus, nccRCC behaves through poorly understood cellular mechanisms (10). In general, nccRCC, especially in the papillary and chromophobe subtypes, have been correlated with a poorer prognosis (11). AAs are four times as likely to have papillary nccRCC and twice as likely to have chromophobe nccRCC relative to their Caucasian counterparts (11). Indeed, AA patients face a myriad of risk factors related to RCC disease epidemiology and social determinants of health that could contribute to their measurably worse outcomes.

Despite the wide adoption of ICIs in real-world settings, there is a paucity of data on differences or similarities experienced by AA and Caucasian mRCC patients with respect to treatment efficacy and safety (2). Durable responses to ICI are seen in only a subset of treated patients, creating a critical need to elucidate the balance of risks and benefits in different racial groups. In this manuscript, we studied ICI outcomes in a real-world patient cohort and analyzed the differences between AA and Caucasian patients with the hope of better informing the use of ICI in AA mRCC patient populations.

## Methods

### Patients and Data Collection

We retrospectively reviewed the clinical outcomes of 198 patients with biopsy-proven diagnoses of mRCC who received at least one dose of ICIs for any line of therapy at the Emory University Winship Cancer Institute from Jan 2015- Jul 2020. A drug administration pharmacy database was used to identify patients. Our cutoff for collecting data was July 12^th^, 2020. Exclusion criteria included incomplete medical records, initiation of ICI at another institution and non-AA or Caucasian racial status, which included 3 patients of Asian descent. Demographic information such as age, gender, disease histology, self-reported race and treatment initiation/termination dates were collected. Additional metrics regarding direct and surrogate measures of clinical efficacy, immune-related adverse events (irAEs) and laboratory data were also collected through the electronic medical records. Responses to therapy were recorded by radiologic evaluation collected at treatment baseline and follow-up appointments. Using computed-tomography scans and magnetic resonance imaging, radiologists at Winship would measure the size of the primary and secondary lesions to gauge the treatment responses after baseline. These findings were later confirmed by study staff using the response evaluation criteria in solid tumors (RECIST) version 1.1.

### Statistical Analysis

Clinical outcomes were measured by overall survival (OS), progression-free survival (PFS), and overall response rate (ORR). OS and PFS were calculated from ICI-initiation to date of death and radiographic or clinical progression, respectively. ORR was defined as the summation of patients who experienced the best radiographic evidence of complete response (CR) or partial response (PR) maintained for at least 6 months per RECIST version 1.1 (12). Statistical analysis was conducted using SAS Version 9.4, and SAS macros developed by Biostatistics Shared Resource at the Winship Cancer Institute (13). The association with OS and PFS was modeled by Cox proportional hazards model and the multivariable models were built by a backward variable selection procedure with an Alpha > 0.2 removal criteria. Univariate associations between each variable and self-identified race was assessed using Chi-square or Fisher’s exact tests for categorical covariates and the ANOVA test for numerical covariates. Univariable and multivariable logistic regression models with the same variable selection strategy were used to estimate odds ratios for ORR.

## Results

### Patients and Tumor Characteristics

Demographic information and baseline disease characteristics for all patients in this cohort are presented in **Table 1**. Our cohort was comprised of 38 AA (19%) and 160 Caucasian (81%) patients (**Table 1**). The median age was 64 years old and the majority of our patients (71%) identified as male. Most of the patients were diagnosed with ccRCC (78%) and more than half received PD-1 monotherapy (57%) with nivolumab. While most patients received ICI monotherapy using a single agent acting through the PD-1/PD-L1 pathway, many of the patients (85) received combination regimens. These consisted of either dual-ICI therapy (n=70) or, amongst a minority of patients in our cohort (n=15), ICI plus a vascular endothelial growth factor (VEGF) inhibitor (**Table 1** **)**. The median number of therapy lines prior to ICI initiation was 1 with 39% of patients having no prior line of therapy. Most patients were international mRCC database consortium (IMDC) intermediate (57%) or poor-risk (25%) groups. The Eastern Cooperative Oncology Groups performance status (ECOG-PS) breakdown for our cohort showed most patients had a score of 1 (46%) or 0 (37%) at ICI initiation. AA patients were significantly more likely to have nccRCC compared to Caucasian patients (41.7% *vs* 17.5% nccRCC, p-0.002). Of note, females constituted 23.8% of the Caucasian group and 50% of the AA group (p=0.002) (**Table 1** **)**.

**Table 1:** Baseline Demographic and Clinical Characteristics of Patients with metastatic RCC by Race.

Covariate	Statistics	Level	Total N=198	Race		P-value*
				Black N=38	White N=160	
Age	Mean		64	61.6	63.2	0.395
	Median		11	62.5	64	
	Std Dev			13.3	10.4	
Gender	N (%)	Female	57 (28.8)	19 (50)	38 (23.8)	**0.001**
	N (%)	Male	141 (71.2)	19 (50)	122 (76.3)	
Non-Clear	N (%)	No	148 (77.9)	21 (58.3)	127 (82.5)	**0.002**
Cell RCC	N (%)	Yes	42 (22.1)	15 (41.7)	27 (17.5)	
Prior Lines (#)	N (%)	0	34 (17.4)	13 (34.2)	64 (40)	0.527
	N (%)	1	83 (41.9)	19 (50)	64 (40)	
	N (%)	2+	38 (19.2)	6 (15.8)	32 (20)	
PD-1 Monotherapy	N (%)	Yes	113 (57)	25 (65.8)	88 (55)	0.472
	N (%)	No (Dual-ICI)	70 (35.4)	11 (28.9)	59 (36.9)	
	N (%)	No (ICI-VEGF)	15 (7.6)	2 (5.3)	13 (8.1)	–
irAEs	N (%)	No	131 (66.2)	29 (76.3)	102 (64.2)	0.153
	N (%)	Yes	66 (33.3)	9 (23.7)	57 (35.8)	
IMDC Risk Group	N (%)	0=Poor	34 (17.4)	4 (10.5)	30 (19.1)	0.354
	N (%)	1=Intermediate	112 (57.4)	22 (57.9)	90 (57.3)	
	N (%)	2=Favorable	49 (25.1)	12 (31.6)	37 (23.6)	
ECOG-PS	N (%)	0	72 (37.3)	7 (19)	64 (41)	–
	N (%)	1	89 (46.1)	19 (51)	70 (45)	
	N (%)	2,3	32 (16.6)	11 (30)	21 (14)	
Best Response	N (%)	CR	9 (4.9)	3 (8.8)	6 (4)	0.06
	N (%)	PR	34 (18.4)	5 (14.7)	29 (19.2)	
	N (%)	SD	57 (30.8)	5 (14.7)	52 (34.4)	
	N (%)	PD	85 (45.9)	21 (61.8)	64 (42.4)	

*The p-value is calculated by ANOVA for numerical covariates; and chi-square test or Fisher’s exact for categorical covariates, where appropriate.

IO, Immunotherapy; PD-L1, Programmed death-ligand 1; RCC, renal cell carcinoma; CC, clear cell; NCC, non-clear cell; IMDC, International Metastatic RCC Database Consortium; ECOG PS, Eastern Cooperative Oncology Groups Performance Status.

**Bold** denotes statistical significance.

### Univariate Analysis of Clinical Efficacy of ICI by Race

**Figure 1:**
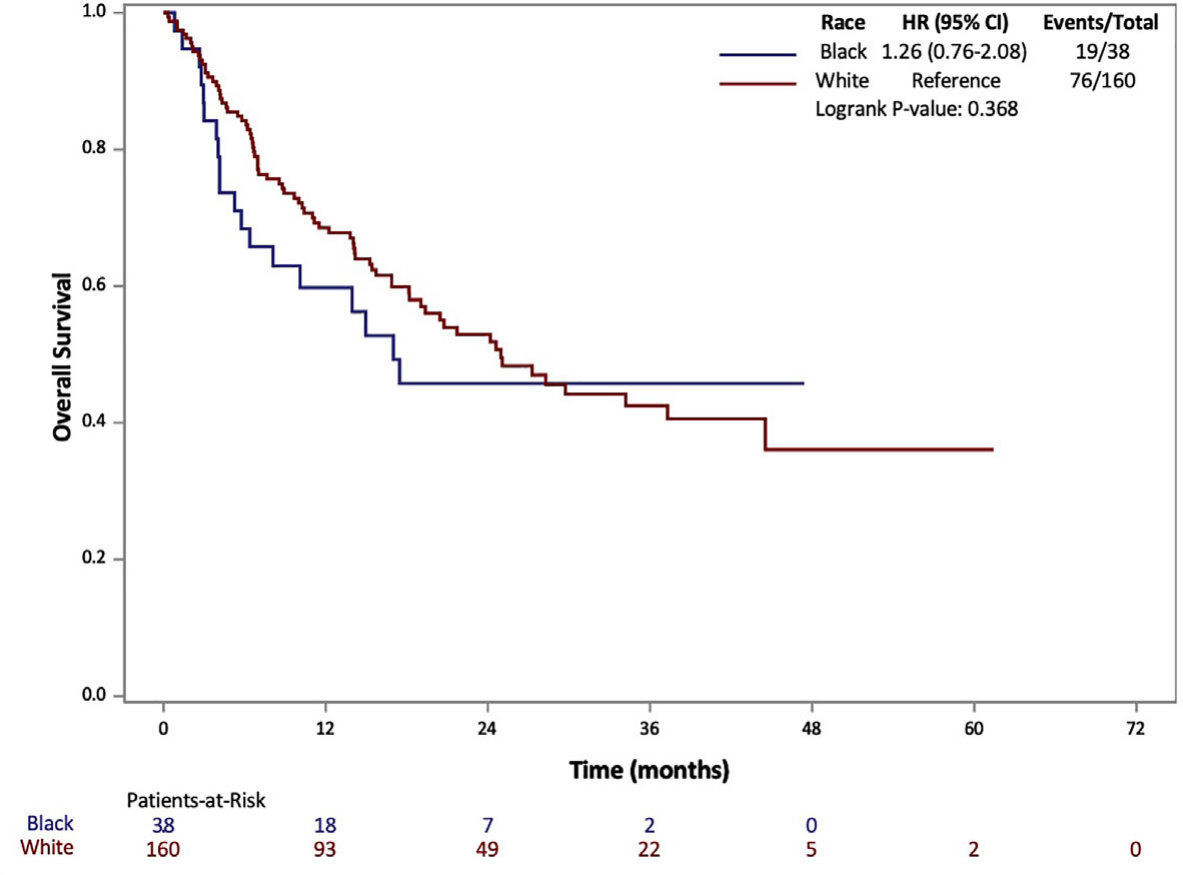
Overall Survival (OS) of patients with metastatic RCC by race: African-American (black) and Caucasian (white).

**Figure 2:**
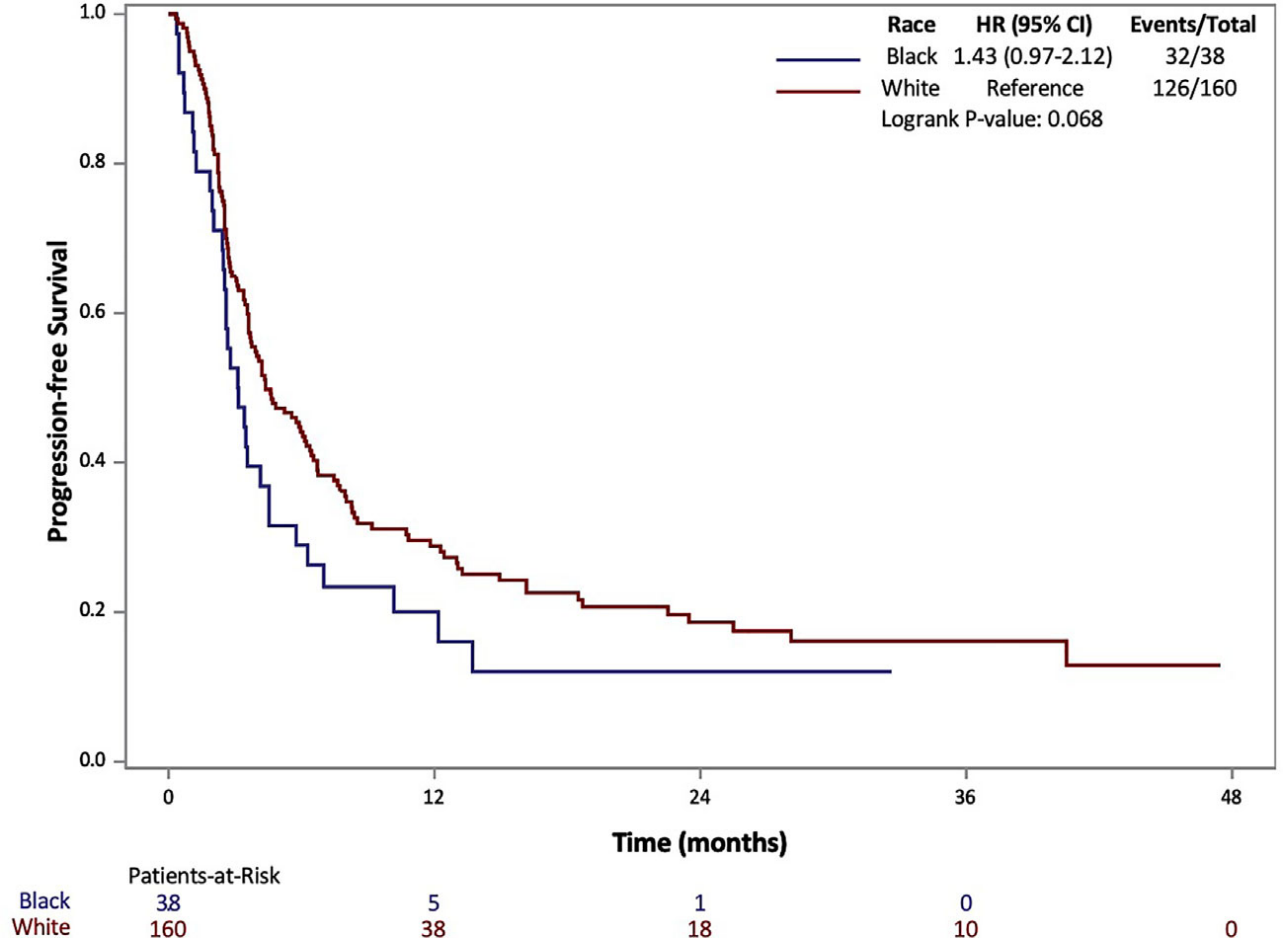
Progression Free Survival (PFS) of patients with metastatic RCC by race: African-American (black) and Caucasian (white).

The results of Kaplan-Meier analysis demonstrated no statistically significant difference for AA patients in median OS (17 *vs* 25 months, p=0.368) compared to Caucasians (**Figure 1**). Similarly, there was no statistically significant difference in median PFS for AA patients compared to Caucasians (3.1 *vs* 4.4 months, p=0.068) (**Figure 2**). Total events and number of patients at risk of events for PFS and OS during the study period are also included in **Figures 1** and **2**. For OS and PFS events, AAs experienced 19/38 and 32/38 respectively. Compared to 76/160 and 126/160 events amongst our Caucasian cohort for OS and PFS respectively.

Both PFS and OS were numerically shorter in AA patients at the 12-month and 24-month marks. In fact, AA patients had a 12-month PFS rate of 20.1% (95% CI: 8.9-34.3%) [*vs*. 28.9% (95% CI: 21.8-36.2%) for Caucasians] and 24-month PFS rate of 12.0% (95% CI: 3.5-26.2%) [*vs*. 18.6% (95% CI: 12.5-25.7%) for Caucasians]. Similarly, AA patients had a 12-month OS rate of 59.8% (95% CI: 42.3-73.5%) [*vs*. 68.5 (95% CI: 60.5-75.3%) for Caucasians] and 24-month OS rate of 45.7% (95% CI: 28.3-61.5%) [*vs*. 52.9 (95% CI: 44.0-61.1%) for Caucasians. Response rates based on radiographic disease surveillance were recorded for the cohort and compared based on self-identified race in UVA (p=0.006). The responses were divided into CR, PR, stable disease (SD) and progressive disease (PD) per RECIST version 1.1. AAs displayed a greater proportion of patients with CR and PD, yet lower rates of PR and SD compared to Caucasian patients. Further details on the rates of treatment responses by race can be found in **Table 1**. AAs also had a numerically lower incidence of irAEs compared to Caucasian patients (23.7% *vs* 64.2%, p=0.153), yet, these findings were not statistically significant. The rates of irAEs predominately consisted of gastro-intestinal (10.7%), endocrine (13.2%) and dermatologic (10.2%) side effects. These rates differed most with irAEs of the endocrine system (2 of 38 AA *vs*. 24 of 160 Caucasian p=0.108) on univariate analysis. More details on irAEs in our cohort can be found in **Supplemental Table 2**.

### Multivariable Analysis of Clinical Efficacy of ICI by Race

AA race was associated with a shorter PFS (HR=1.52, 95% CI: 1.01-2.3, p=0.045) on multivariable analysis (**Table 2**). Higher IMDC risk score and a greater number of prior therapies also predicted worse PFS on multivariable analysis. Interestingly, race was not associated with differences in OS under univariate and multivariate analysis of clinical characteristics. As with the PFS analysis, higher IMDC risk group and prior lines of therapy were associated with worse OS (**Table 3**). AA race was associated with a similar ORR (OR=1.04, 95% CI: 0.42-2.57, p=0.936) after controlling for age, race, gender, IMDC risk group, number of prior lines of therapy, PD-1 monotherapy and ccRCC in MVA (**Supplemental Table 1** **)**.

**Table 2:** Univariate and Multivariate Association between PFS and Clinical Characteristics in Patients with Metastatic RCC.

Covariate	Level	N	Progression Free Survival					
			Univariate Analysis			Multivariate Analysis		
			Hazard Ratio (95% CI)	HR P-value	P-value	Hazard Ratio (95% CI)	HR P-value	P-value
Race	Black	38	1.43 (0.97-2.12)	0.07	0.068	1.50 (1.01-2.32)	**0.048**	**0.048**
	White	160	–	-		–	–	
Gender	Female	57	0.87 (0.61-1.22)	0.412	0.41	0.75 (0.52-1.08)	0.123	0.123
	Male	141	–	-		–	–	
Non-Clear Cell RCC	Yes	42	1.12 (0.76-1.64)	0.581	0.58	–	–	–
	No	148	–	-		–	–	
PD-1 Monotherapy	Yes	113	1.38 (1.00-1.91)	0.051	**0.049**	–	–	–
	No	85	–	-		–	–	
IMDC Risk Group	0=Poor	34	–	-	**0.002**	–	-	0.008
	1=Intermediate	112	2.05 (1.28-3.31)	**0.003**		1.87 (1.16-3.02)	**0.01**	
	2=Favorable	49	2.47 (1.45-4.19)	**<.001**		2.33 (1.35-4.01)	**0.002**	
Prior Lines (#)	0	77	–	-	**<.001**	–	-	**0.001**
	1	83	1.17 (0.81-1.67)	0.397		1.20 (0.83-1.74)	0.325	
	2+	38	2.32 (1.52-3.54)	**<.001**		2.22 (1.43-3.43)	**<.001**	
Age		198	0.99 (0.98-1.01)	0.299	0.299	–	–	–

*The p-value is calculated by ANOVA for numerical covariates; and chi-square test or Fisher’s exact for categorical covariates, where appropriate.

IO, Immunotherapy; PD-L1, Programmed death-ligand 1; RCC, renal cell carcinoma; CC, clear cell; NCC, non-clear cell; IMDC, International Metastatic RCC Database Consortium; ECOG PS, Eastern Cooperative Oncology Groups Performance Status.

**Bold** denotes statistical significance.

**Table 3:** Univariate and Multivariate Association between OS and Clinical Characteristics in Patients with Metastatic RCC.

Covariate	Level	N	Overall Survival					
			Univariate Analysis			Multivariate Analysis		
			Hazard Ratio (95% CI)	HR P-value	P-value	Hazard Ratio (95% CI)	HR P-value	P-value
Race	Black	38	1.26 (0.76-2.08)	0.369	0.368	1.02 (0.57-1.84)	0.947	
	White	160	–	-		–	–	0.947
Gender	Female	57	0.77 (0.49-1.21)	0.255	0.253	0.60 (0.35-1.02)	0.061	
	Male	141	–	-		–	–	0.061
Non-Clear Cell RCC	Yes	42	1.68 (1.05-2.69)	**0.031**	**0.029**	1.56 (0.95-2.55)	0.078	
	No	148	–	-		–	–	0.078
PD-1 Monotherapy	Yes	113	1.43 (0.93-2.21)	0.107	0.105	–	–	**-**
	No	85	–	-		–	–	
IMDC Risk Group	0=Poor	34	–	-	**<.001**	–	–	**0.001**
	1=Intermediate	112	2.14 (1.05-4.36)	**0.037**		1.80 (0.88-3.69)	0.11	
	2=Favorable	49	4.93 (2.36-10.33)	**<.001**		4.38 (2.03-9.44)	**<.001**	
Prior Lines (#)	0	77	–	-	**0.001**	–	–	0.011
	1	83	1.18 (0.72-1.94)	0.51		1.21 (0.71-2.05)	0.486	
	2+	38	2.43 (1.43-4.13)	**0.001**		2.10 (1.19-3.71)	**0.011**	
Age		198	198	1.00 (0.98-1.02)	0.743	–	–	–

*The p-value is calculated by ANOVA for numerical covariates; and chi-square test or Fisher’s exact for categorical covariates, where appropriate.

IO, Immunotherapy; PD-L1, Programmed death-ligand 1; RCC, renal cell carcinoma; CC, clear cell; NCC, non-clear cell; IMDC, International Metastatic RCC Database Consortium; ECOG PS, Eastern Cooperative Oncology Groups Performance Status.

**Bold** denotes statistical significance.

## Discussion

In our study of clinical outcomes for patients with mRCC, we found similar efficacy (median OS and PFS) and safety (incidence of irAEs) profiles for ICI therapy when comparing self-identified racial groups. AA race was associated with shorter PFS with no difference in OS compared to Caucasian patients after controlling for confounders such as age, RCC histology and gender. These observations are most plausibly due to a multifactorial cause, and, in this discussion, we will highlight some potential contributors to these differences. Nonetheless, our study displays reassuring outcomes data for the use of ICI therapy in real-world patient populations.

AAs comprised 10% of total RCC diagnosis from 2001-2010 (14). Our study cohort, composed of nearly 20% AAs, offers an analysis of clinical outcomes that can better represent the efficacy and safety of ICIs with AA patients. To our best knowledge, the outcomes analysis in this study contains the largest percentage of AA mRCC patients treated with ICI therapy to date. Our results better represent the patient outcomes for AAs in the real-world setting when compared to other available studies of mRCC and ICI. Most notably, we included all patients at our center with RCC who received at least one dose of ICI. This provided a more generalizable sample relative to the real-world patient population we hoped to emulate. This representative cohort included patients who were often less healthy and more diverse than the samples used in clinical trials (15). Of note, patients with an ECOG-PS of 2 or more made up only 8% of the study cohorts in phase III clinical trials (16). Meanwhile, our cohort included nearly twice as many as a percentage of total with more than 16% of our patients having an ECOG greater than 2.

The epidemiology of renal malignancies have a long history of racial disparities and researchers have begun to better quantify these disparate outcomes in the past decades (17). One factor often cited in RCC disparity research is the epidemiology of histologic phenotypes that are crucial for cancer diagnostics and prognostication. NccRCC specifically has shown limited efficacy with newer treatment modalities such as ICIs, and this diagnosis has a much greater prevalence in AA patient populations compared to Caucasian patients. Additionally, clinical trials studying RCC patients predominately study outcomes in ccRCC patients (10). Taken together, nccRCC’s ill-defined histology, aggressive phenotype and limited therapy options makes it carry a poor prognosis compared to ccRCC. This is especially relevant in the age of targeted and pathway specific therapy, as these cellular diagnostics are becoming integral to the management of disease. In our cohort, AA patients displayed significantly higher rates of nccRCC. This is notable to mention because including a disproportionately large number of AA patients with nccRCC could skew the AA cohort towards worse outcomes on univariable analysis. However, even after controlling for cancer histology, we still found that AA race was associated with significantly shorter PFS compared to Caucasians. Additionally, while shorter PFS for AA patients was the only statistically significant difference on MVA, our AA cohort also displayed measurably shorter median OS, median PFS and ORR. We will attempt to highlight potential contributors for these disparities in the remainder of our discussion. However, put simply, we believe the difference noted in our analysis and from the RCC racial disparities literature can be largely attributed to a multifactorial etiology of socio-economic forces that impact the outcomes and access to care experienced by AA patients with oncologic disease. Nevertheless, these numeric differences did not translate into a significant difference for OS, which is noteworthy as an encouraging finding for ICI usage in the real-world setting.

Within the field of immuno-oncology, non-trivial differences are found amongst different racial groups in the way the immune system manages cancer (18). Researchers postulate that alterations in the stress response from the hypothalamic-pituitary-adrenal (HPA) axis, leading to systemic hormonal changes, can impair immune-related functions and cause decreased tumor clearance amongst certain groups of patients (19). *Neighborhood physical disorder* is a condition often cited in bio-psychosocial models that links societal and systemic stressors to chronic inflammation which can drive immune dysregulation and poor health outcomes amongst disadvantaged communities (20). Additionally, researchers have also considered the disproportionate rates of vitamin D deficiency amongst African Americans as another potential contributor to healthcare disparities and sub-optimal immune function in this population (21). Put simply, we feel it is important to identify the potential differences in cancer biology amongst racial groups because it could be useful in the application of IO therapy in minority populations with oncologic disease. That being said, while these biologic differences were historically cited in the oncology literature to explain racially-based disparities, we agree with a growing body of evidence that highlights the considerable impact that social, economic and healthcare-access issues play in the racial disparities of cancer patients (22, 23). It is imperative that oncologists appreciate how historical and sociopolitical forces intertwine with race because of the insidious impacts they can have on patients managing complicated disease such as mRCC (24). The findings from our cohort are supported by the current stance within the racial disparities research of RCC and add specificity, primarily in PFS, to how clinical outcomes could differ with the use of immune acting therapies in AAs and Caucasian patients. These differences are likely due to a multifactorial etiology that stem from a combination of biological and societal factors.

The literature’s stance on race and immune-related adverse events (irAE) is still developing; however, some studies have found minority groups, specifically AAs, experience lower rates of irAEs relative to Caucasian patients (25). Taking these immune toxicity rates into account, there is a possibility that these racial differences in the immune system could impact the function of immunotherapy in minority patients. Within our cohort, we found no statistically significant difference in the safety profile of ICI, yet a much lower incidence of irAE in AA patients (23.7%) compared to Caucasians (64.2%). This difference could become more (or less) pronounced upon studying a larger cohort.

Given the explosive rise of ICI therapy in the treatment of mRCC, it is important to appreciate the interplay of biologic and systemic contributors in the efficacy and safety of ICI utilization with AA patients. Overall, our study provides evidence that clinical outcomes are mostly comparable between AA and Caucasian patients managed on ICI. We found no differences on the Kaplan-Meir level, but did note an association of AA race with worse PFS on multivariable analysis. We hypothesize that the latter could be due to factors such as unmeasured comorbidities and complex social determinants of health. Despite this difference in PFS, our findings support an imperative notion within disparities research that equal treatment provided to equal patients, regardless of race, should result in similar outcomes. However, the presence of racial disparities within the literature displays the need for further research in this field to delineate the medical and socioeconomic factors that cause these population-level outcome inequities.

The limitations of this study include the smaller overall size of our cohort and the binary racial categories used. This is relevant since the racial disparities research within RCC has also attributed poorer outcomes to Hispanic and Native/Alaskan American populations (26). Another limitation is our lack of sociodemographic data on our cohort such as the income level of patients. While all patients included in this study had health insurance, they were not differentiated on the basis of private or public provision. We also used a retrospective study from a single cancer institute, which is subject to selection bias. However, we attempted to mitigate this concern by including all patients who received one dose of ICI regardless of histology or other disease-specific characteristics. While our inclusion criteria allowed us to collect a larger number of patients, there was some degree of heterogeneity for the different ICI therapy options patients could receive. This included IO-monotherapy, IO-dual therapy and IO-TKI combination therapy. The rates of dual *vs* mono-IO therapy can be seen in **Table 1**. Our findings in **Table 1** also show that AAs were more likely to receive monotherapy instead of combination therapy (65% *vs* 55%), and to be of a higher ECOG-PS 2-3 (30% *vs* 14%). Additionally, many patients did not receive these IO regimens as first-line therapy and our data displayed worsening prognosis as patients had more lines of prior therapy. This degree of variance between treatment approaches is commonplace in this type of real-world analysis and allows the results of our study to better emulate the expected effect of immunotherapy in practice. Since not every patient in our cohort was able to receive cancer genetic testing or mutation profiles, we chose not to include biomarkers of ICI response such as PD-L1 expression. Further, our secondary clinical outcome, overall response rate or ORR, is not standard in clinical trials. Larger datasets are needed to investigate the statistically significant findings in the current study, namely the association of shorter PFS and AA race on MVA.

Despite its limitations, we believe our current study has numerous strengths. Our patient sample was drawn from a single cancer institute and, therefore, represents a homogenous population in terms of geographic residence and access to cancer care in the United States. In our multivariable analysis, many demographic and clinical factors specific to our patients were controlled for.

The treatment landscape of RCC continues to evolve as more therapy options become available to patients, specifically ICI-VEGF TKI combinations. During our study period from 2015-2020, less than half of our patients (n=85) (**Table 1** **)** in the cohort received combination ICI-TKI or dual-ICI therapy. This is a lower proportion of patients than would have received combination therapy today in light of the FDA approvals for combination regimens in mRCC: nivolumab + ipilmumab (April 2018), pembrolizumab + axitinib (April 2019), avelumab + axitinib (May 2019) nivolumab + cabozantinib (January 2021) and lenvatinib + pembrolizumab (April 2021) (27, 28). We note that two contemporary analyses of mRCC patients treated with TKI showed that race (AA *vs* Caucasian) was not independently associated with differing survival outcomes (29, 30). Similar to our study, the comparable OS between AA and Caucasians are encouraging findings for the use of mono and combination immunotherapy in AA mRCC patient populations.

## Conclusion

In our cohort, we analyzed clinical outcomes amongst mRCC patients treated on ICI therapy. Overall, our study suggested a favorable benefit-to-risk ratio of ICI for the treatment of mRCC in AA patients. We found comparable outcomes for AA and Caucasian patients for OS, median PFS, ORR and immune-related adverse events. Our multivariable analysis of outcomes showed an association of AA race with shorter PFS that warrants additional investigation. Larger prospective studies from multiple institutions are needed to validate these findings, especially amongst other non-AA US minority populations. We hope our real-world data may help oncologic physicians appreciate a degree of nuance when treating increasingly diverse mRCC patients and emphasize the need for improved inclusion criteria for racial minority groups in future IO clinical trials.

## Data Availability Statement

The raw data supporting the conclusions of this article will be made available by the authors, without undue reservation.

## Ethics Statement 

The study was approved by the Emory University Institutional Review Board and was conducted in accordance with Good Clinical Practice Guidelines and the Declaration of Helsinki. The patients/participants provided their written informed consent to participate in this study.

## Author Contributions 

TO was involved in collecting references as well as writing and editing the manuscript. DM was involved in the data acquisition and administrative support. BN and MAB provided advisory on the manuscript and final revisions prior to submission. YL and SG provided assistance with statistical analysis. All remaining authors were involved in the care of the patients in this study, interpretation and analysis of study results, and editing of the manuscript. All authors contributed to the article and approved the submitted version.

## Funding

Research reported in this publication was supported in part by the Breen Foundation and the Biostatistics Shared Resource of Winship Cancer Institute of Emory University and NIH/NCI under award number P30CA138292. The content is solely the responsibility of the authors and does not necessarily represent the official views of the National Institutes of Health.

## Conflict of Interest

MB has acted as a paid consultant for and/or as a member of the advisory boards of Exelixis, Bayer, BMS, Eisai, Pfizer, AstraZeneca, Janssen, Genomic Health, Nektar, and Sanofi and has received grants to his institution from Xencor, Bayer, Bristol-Myers Squibb, Genentech/Roche, Seattle Genetics, Incyte, Nektar, AstraZeneca, Tricon Pharmaceuticals, Peleton Therapeutics, and Pfizer for work performed as outside of the current study. BN has acted as a paid member of the advisory board of Exelixis.

The remaining authors declare that the research was conducted in the absence of any commercial or financial relationships that could be construed as a potential conflict of interest.
