# Supplementary material for: Racial Differences in Clinical Outcomes for Metastatic Renal Cell Carcinoma Patients Treated With Immune-Checkpoint Blockade
Source: Front Oncol. 2021 Jun 16;11:701345. doi: 10.3389/fonc.2021.701345 (PMC8242950; doi:10.3389/fonc.2021.701345)
Supplement: Supplementary file 1 [file Table_1.pdf]

Supplemental Table 1: Univariate and Multivariate Association between ORR and Clinical Characteristics in Patients with Metastatic RCC

Covariate	Level	N	Overall Response Rate					
			Univariate Analysis			Multivariate Analysis		
			Odds Ratio (95% CI)	OR P-value	Overall P-value	Odds Ratio (95% CI)	OR P-value	Overall P-value
Race	Black	34	1.02 (0.42-2.45)	0.965	0.965	1.04 (0.42-2.57)	0.936	0.936
	White	151	-	-		-	-	
Gender	Female	54	1.07 (0.51-2.25)	0.864	0.864	0.53 (0.21-1.31)	0.169	0.169
	Male	131	-	-		-	-	
Non-Clear Cell RCC	Yes	38	1.19 (0.52-2.72)	0.673	0.673	1.13 (0.45-2.82)	0.8	0.109
	No	139	-	-		0.22 (0.04-1.13)	0.069	
						-	-	-
PD-1 Monotherapy	Yes	106	0.44 (0.22-0.88)	**0.021**	**0.021**	-	-	-
	No	79	-	-		-	-	-
						-	-	-
IMDC Risk Group	1	106	0.91 (0.38-2.20)	0.837	0.428	-	-	-
	2	43	0.52 (0.17-1.58)	0.248		-	-	-
	0	33	-	-		-	-	-
						-	-	-
Prior Iines (#)	1	77	0.76 (0.37-1.56)	0.454	**0.039**	-	-	-
	2+	35	0.14 (0.03-0.64)	**0.011**		-	-	-
	0	73	-	-		-	-	-
						-	-	-
Age		185	0.99 (0.96-1.02)	0.537	0.537	-	-	-

Supplemental Table 2: Univariate Association between irAEs and self-identified race

Covariate	Level	Race		P-value*
		Black N=38	White N=160	
Immune-Related Adverse Events	No	29 (76.3)	102 (64.2)	0.153
	Yes	9 (23.7)	57 (35.8)	
Pulmonary irAE	No	37 (97.4)	154 (96.9)	1
	Yes	1 (2.6)	5 (3.1)	
Dermatologic irAE	No	35 (92.1)	142 (89.3)	0.77
	Yes	3 (7.9)	17 (10.7)	
Endocrine irAE	No	36 (94.7)	135 (84.9)	0.108
	Yes	2 (5.3)	24 (15.1)	
Thyroid irAE	No	37 (97.4)	143 (89.9)	0.203
	Yes	1 (2.6)	16 (10.1)	
Adrenal irAE	No	37 (97.4)	152 (95.6)	1
	Yes	1 (2.6)	7 (4.4)	
Pituitary irAE	No	38 (100)	155 (97.5)	1
	Yes	0 (0)	4 (2.5)	
Renal irAE	No	37 (97.4)	153 (96.2)	1
	Yes	1 (2.6)	6 (3.8)	
MSK irAE	No	36 (94.7)	153 (96.2)	0.652
	Yes	2 (5.3)	6 (3.8)	
GI irAE	No	36 (94.7)	140 (88.1)	0.379
	Yes	2 (5.3)	19 (11.9)	
Hepatic irAE	No	36 (94.7)	147 (92.5)	1
	Yes	2 (5.3)	12 (7.5)	
Pancreatic irAE	No	38 (100)	154 (96.9)	0.585
	Yes	0 (0)	5 (3.1)	
Colitis	No	38 (100)	157 (98.7)	1
	Yes	0 (0)	2 (1.3)	
Other irAE	No	38 (100)	155 (97.5)	1
	Yes	0 (0)	4 (2.5)	
